# Free Sugars and Total Fat Are Important Characteristics of a Dietary Pattern Associated with Adiposity across Childhood and Adolescence^1^,^2^,^3^

**DOI:** 10.3945/jn.115.224659

**Published:** 2016-03-09

**Authors:** Gina L Ambrosini, David J Johns, Kate Northstone, Pauline M Emmett, Susan A Jebb

**Affiliations:** 4 School of Population Health, The University of Western Australia, Perth, Australia;; 5 Medical Research Council Human Nutrition Research, Cambridge, United Kingdom;; 6 Public Health Directorate, National Health Service Lincolnshire, Lincoln, United Kingdom;; 7 School of Social and Community Medicine, University of Bristol, Bristol, United Kingdom; and; 8 Nuffield Department of Primary Care Health Sciences, University of Oxford, Oxford, United Kingdom

**Keywords:** children, adolescents, obesity, adiposity, diet, dietary patterns, sugar, fat, energy density, ALSPAC

## Abstract

**Background:** The importance of dietary sugar compared with fat in the development of obesity is currently a topic of debate.

**Objective:** We aimed to identify dietary patterns (DPs) characterized by high sugar content, high fat content, or both and their longitudinal associations with adiposity during childhood and adolescence.

**Methods:** Participants were 6722 children from the ALSPAC (Avon Longitudinal Study of Parents and Children) who were born in 1991–1992. DPs were characterized by percentage of total energy intake (%E) from free sugars, %E from total fat, and dietary energy density (DED) and fiber density by using reduced rank regression at 7, 10, and 13 y of age. Total body fat mass was measured at 11, 13, and 15 y of age. Regression analyses were used to adjust for dietary misreporting, physical activity, and maternal social class.

**Results:** Two major DPs were identified: higher *z* scores for DP1 were associated with greater DED, greater %E from free sugars and total fat, and lower fiber density; higher *z* scores for DP2 were associated with greater %E from free sugars but lower %E from total fat and DED. A 1-SD increase in *z* score for DP1 was associated with a mean increase in the fat mass index *z* score of 0.04 SD units (95% CI: 0.01, 0.07; *P* = 0.017) and greater odds of excess adiposity (OR: 1.12; 95% CI: 1.0, 1.25; *P* = 0.038). DP2 was not associated with adiposity.

**Conclusions:** An energy-dense DP high in %E from total fat and free sugars is associated with greater adiposity in childhood and adolescence. This appears to confirm the role of both fat and sugar and provides a basis for food-based dietary guidelines to prevent obesity in children.

## Introduction

Overweight and obesity in childhood and adolescence are widely acknowledged to be a serious and pressing public health concern. Excess weight gain during childhood is associated with adverse cardiometabolic profiles as well as other disorders (1), which tend to persist over time and into adulthood (2).

Recently, there has been renewed debate about the dietary determinants of obesity, with suggestions that sugar is a more important risk factor than dietary fat (3). There is evidence to suggest that both are causes for concern, because both are associated with the overconsumption of energy (4). A meta-analysis of randomized controlled trials and observational studies showed that high-fat diets are associated with greater relative body weight in adults and children (5). Another meta-analysis concluded that the addition of sugar to the diet increases body weight (6), and the WHO recently published guidelines recommending reducing the intake of free sugars to <10% of total energy intakes (EIs)^9^ across the life course (7). However, in practice, consumers make decisions about foods rather than individual nutrients. Few foods are composed solely of fat or sugar, and the combination of sugar with fat creates a “hedonic synergy” (4) underpinning the high palatability of foods such as cakes, biscuits, and chocolate (8), which can undermine innate appetite control. To develop practical guidance for consumers to reduce the risk of excess weight gain, it may be more helpful to consider the relation between whole foods or overall dietary patterns (DPs) and the risk of obesity.

In 2012 we identified an energy-dense, high-fat, low-fiber DP longitudinally associated with increased fat mass index (FMI) and excess adiposity between 7 and 15 y of age in a large United Kingdom birth cohort (9). We chose a priori to investigate an empirically derived DP characterized by these nutrients, because earlier WHO reviews supported fiber and energy density as convincing dietary risk factors for obesity and dietary fat as a major contributor to dietary energy density (DED) (10). Apart from sugar-sweetened beverages (SSBs), there was less convincing evidence for an association between free sugar intake and obesity at that time (10). Because of the recent emphasis on free (or added) sugars in the diet as an additional risk factor for weight gain (6) and obesity, here we extend our 2012 analysis by investigating DPs characterized by free sugars in addition to energy density, fat, and fiber, which, to our knowledge, has not previously been done in this cohort. We hypothesized that a high-sugar DP would be more strongly associated with body fatness than our original high-fat, energy-dense, low-fiber DP (9).

## Methods

### 

#### Study population.

Data were sourced from the Avon Longitudinal Study of Parents and Children (ALSPAC), which has been described in full elsewhere (11). Briefly, ALSPAC is an observational birth cohort that recruited 14,541 pregnant women in Avon, England, with an expected delivery date between 1 April 1991 and 31 December 1992. Of the 14,472 known birth outcomes, 14,062 were live births and 13,988 were alive at 1 y. An additional 713 children whose mothers were initially invited but had not enrolled were recruited later. The total baseline cohort therefore included 14,701 children who were alive at 1 y. Data have been collected regularly from the children and their families via questionnaires and clinic visits (11). Data from follow-ups of index offspring at 7 y (1998–2000; *n* = 8297), 10 y (2002–2003; *n* = 7563), 11 y (2003–2005; *n* = 7159), 13 y (2005–2006; *n* = 6147), and 15 y (2006–2008; *n* = 5509) of age were used in the present analysis. The study website contains details of all of the data that are available through a fully searchable data dictionary (12). Parents provided written consent for their child to participate in the study. Ethical approval for the study was obtained from the ALSPAC Law and Ethics Committee and the local research ethics committees.

#### Anthropometric measurements.

DXA was used to measure total body fat mass (FM) at the 11-, 13-, and 15-y follow-ups with the use of a Lunar Prodigy DXA fan beam scanner (GE Medical Systems Lunar). As previously described (9), we calculated the FMI separately for boys and girls and at each follow-up visit by dividing fat mass (kg) by height (m) raised to the optimum power [calculated by using log-log regression analysis (13)] to remove any residual correlation between FM and height (i.e., FM/height*^x^*). The FMI was then log-transformed to obtain normal distributions and standardized to a *z* score to support comparisons. Excess adiposity was identified as being in the top quintile (>80th percentile) of FMI *z* scores. Weight was measured to the nearest 0.1 g at each follow-up visit by using a Tanita body fat analyzer (Tanita Corporation). Height was measured to the nearest 0.1 cm without shoes and socks by using a Harpenden stadiometer (Holtain Ltd.).

#### Dietary assessments.

Three-day unweighed food diaries were completed at 7 y of age by the parent and at 10 and 13 y of age by the study child, with parental assistance. The food diary was recorded over 3 nonconsecutive days (2 weekdays and 1 weekend day) and completed diaries were checked and queried with the child and parent by a nutritionist at clinic visits. The diaries were coded by using DIDO (14), a program developed at the Medical Research Council Human Nutrition Research Unit, Cambridge, United Kingdom, and linked with British food-composition tables to estimate mean daily nutrient intakes.

The intake of free sugars was estimated as “all monosaccharides and disaccharides added to foods by the manufacturer, cook or consumer, plus sugars naturally present in honey, syrups, fruit juices and fruit juice concentrates,” as defined by the WHO (7). Food intakes recorded in the 3-d food diaries were categorized into 46 major food groups as used in previous analyses of ALSPAC dietary data (9, 15). Dietary misreporting was estimated by using the ratio of EI to estimated energy requirement (EER). A 95% CI was calculated to classify individuals as plausible reporters, overreporters (EI:EER >95% CI) or underreporters (EI:EER <95% CI) (16).

#### DPs.

As per our 2012 DP analysis in this cohort (9), we used reduced rank regression (RRR) to identify DPs, or combinations of food intake, that attempt to explain the maximum variation in a set of response variables hypothesized to be on the pathway between food intake and obesity. The RRR model included intakes of all 46 food groups (g/d) as predictor variables and the following 4 response variables: the proportion of total energy (%E) from free sugars (%E from sugar), the %E from total fat (%E from fat), DED as energy (kJ) per gram of food consumed [excluding beverages (17)], and dietary fiber density (FD) as grams of nonstarch polysaccharide fiber per megajoule of total energy. The RRR model extracts successive linear combinations of food intakes (factors or DPs) until they explain the maximum amount of shared variation in all response variables (18). The final number of DPs is equal to the number of response variables in the model, and the first DP typically explains the most shared variation among all response variables, with subsequent patterns explaining smaller proportions of the remaining variation. Respondents were scored for each DP at each age with the use of a *z* score that quantified how their reported dietary intake reflected each DP relative to other respondents in the study sample. The RRR model calculates DP *z* scores for each respondent as a linear, weighted combination of all of their standardized food group intakes by using weights unique to each DP. Increasing intakes of foods with positive factor loadings increases the DP *z* score; increasing intakes of foods with negative factor loadings decreases the DP *z* score.

The first 2 DPs (DP1 and DP2) derived in the RRR analysis consistently explained the greatest amount of shared variation in all response variables (24–35% each) at 7, 10, and 13 y (**Supplemental Table 1**). The remaining 2 DPs (DP3 and DP4) explained relatively little shared variation (5–8% each) and were therefore not taken forward for further investigation. The foods characterizing DP1 and DP2 and the amount of variation they explained for individual response variables were also similar across ages (note: DP2 at 10 y was the exact inverse of DP2 observed at 7 and 13 y; Supplemental Table 1) and did not vary substantially by sex (data not shown). However, to assess longitudinal changes in *z* scores for exactly the same DPs (those identified at 7 y), confirmatory RRR was applied to calculate *z* scores at 10 and 13 y of age by using the scoring weights identified at 7 y of age.

#### Physical activity and other variables.

Total physical activity was measured with the use of an MTI Actigraph AM7164 2.2 accelerometer at 11 and 13 y of age and analyzed as counts per minute (logged) averaged over 3 d (Actigraph LLC) (19). Pubertal status was self-reported at 11 and 13 y by using diagrams depicting the 5 Tanner stages for pubic hair development (20). Maternal social class, classified from I (professional) to IV (semiskilled) and V (unskilled manual workers) and based on most recent or current occupation, was assessed by questionnaire at 32 wk of gestation.

#### Statistical analyses.

Because nutrient intakes were normally distributed, mean (±SD) intakes were estimated by year of follow-up to describe intakes in the study population. Mean nutrient intakes across increasing quintiles of DP1 and DP2 *z* scores were estimated by using linear regression. A trend in mean nutrient intake across increasing quintiles was tested by modeling DP quintiles as continuous variables and by using a *z*-test to test the null hypothesis (mean change in nutrient intake per quintile increase = 0).

As previously described (9) and to make use of all available dietary and adiposity data, generalized estimating equations (GEEs) were applied to investigate longitudinal associations between DP *z* scores and FMI *z* scores. These models regressed FMI on DP *z* score at the previous time point by using DP *z* scores at 7, 10, and 13 y of age and FMI *z* scores at 11, 13, and 15 y of age. A GEE model was similarly applied to examine the odds of excess adiposity in relation to DP *z* score. All GEE models were run by using the “xtgee” command in Stata with an exchangeable correlation structure. Because the DPs produced by RRR are uncorrelated (orthogonal) (18), *z* scores for DP1 and DP2 were analyzed in mutually adjusted models to examine their independent associations with adiposity. Models tested time-varying covariates (i.e., age, dietary misreporting, physical activity, Tanner stage) and fixed covariates (sex, maternal social class). Scores for DP1 and DP2 were modeled as continuous (*z* scores) and categorical (quintiles) variables. We tested for trends in associations between the outcomes and increasing DP quintiles by modeling DP quintiles as continuous variables. All GEE models used the *z*-test to test the null hypothesis that each β-coefficient was equal to zero. An α level ≤0.05 was used in all analyses. No interactions between DPs and sex were observed and so boys and girls were analyzed together, including sex as a covariate. To test if the strength of association (if any) between the DPs and adiposity outcome varied by age, an interaction term between DP *z* score and age at dietary assessment was tested in each model. The Stata version 13 (I/C for Windows) code created for this statistical analysis is available from the corresponding author upon request.

## Results

A description of the study population has been published previously (9). Food diary data were available for 7285, 7471, and 6106 children at 7, 10, and 13 y of age, respectively. Mean daily intakes of key nutrients and anthropometric measurements are shown in **Supplemental Table 2**. Intakes of %E from sugar were high, averaging between 17.4% (median: 17%) at 7 y and 16.6% (median: 16%) at 13 y of age. The mean %E from fat at each follow up was ∼35% or more (Supplemental Table 2).

**FIGURE 1:**
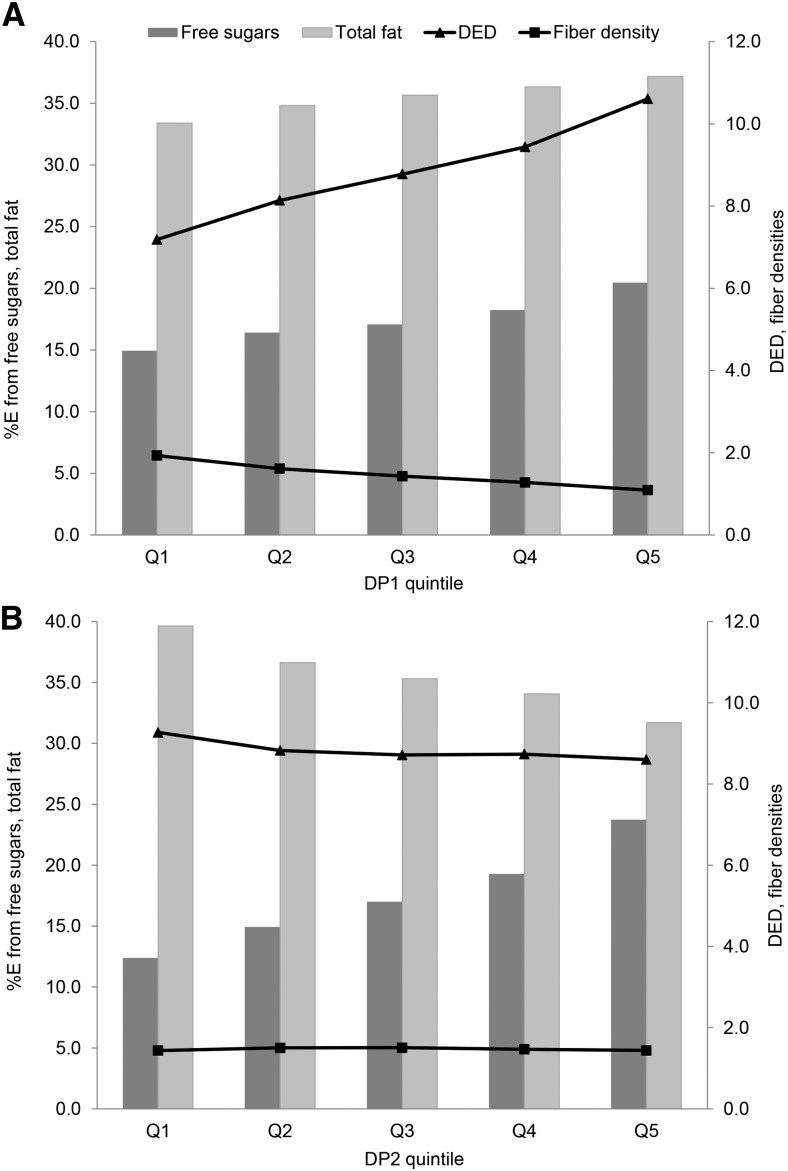
Intakes of response variables by quintile of DP *z* scores characterized by %E from free sugars, %E from total fat, DED (in MJ · g^−1^ · d^−1^), and fiber density (in g · MJ^−1^ · d^−1^) at 7 y of age in the ALSPAC cohort for DP1 (A) and DP2 (B). Values are means, *n* = 1457 in each quintile. ALSPAC, Avon Longitudinal Study of Parents and Children; DED, dietary energy density; DP, dietary pattern; DP1, energy-dense, high %E from free sugars, high %E from total fat, low fiber dietary pattern; DP2, non–energy dense, high %E from free sugars, and low %E from total fat dietary pattern; Q, quintile; %E, proportion of total energy intake.

**FIGURE 2:**
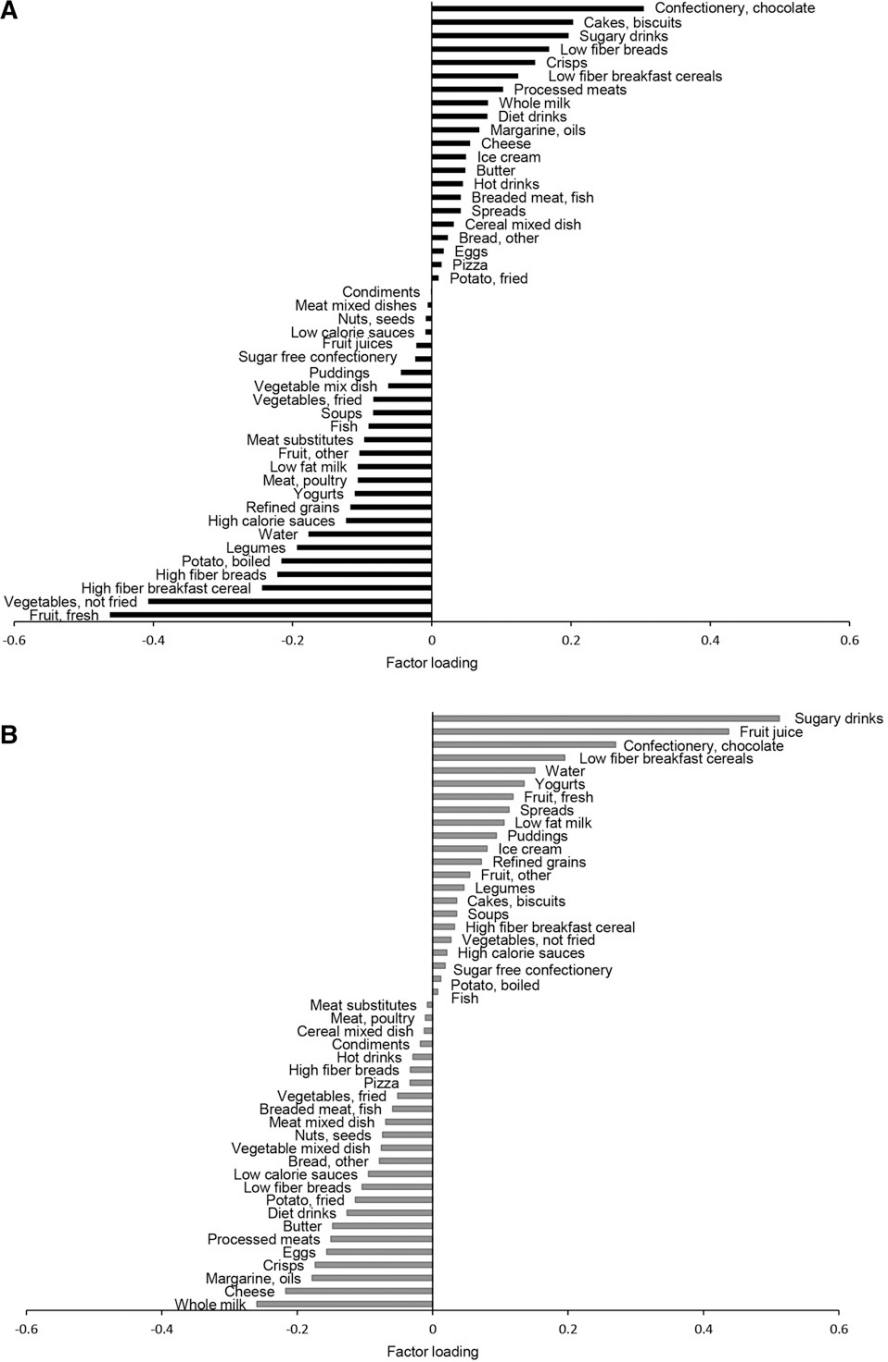
Factor loadings for dietary patterns characterized by %E from free sugars, %E from total fat, dietary energy density, and fiber density calculated by using reduced rank regression at 7 y of age in the ALSPAC cohort for DP1 (A) and DP2 (B). ALSPAC, Avon Longitudinal Study of Parents and Children; DP1, energy-dense, high %E from free sugars, high %E from total fat, low-fiber dietary pattern; DP2, non–energy dense, high %E from free sugars, and low %E from total fat dietary pattern; %E, proportion of total energy intake.

At 7 y of age, the first major DP (DP1) correlated positively with DED, %E from fat, and %E from sugar and negatively with FD, as indicated by their respective response variable weights: 0.67, 0.26, 0.31, and −0.63 (Supplemental Table 1). DP1 explained a majority of the variation in DED (63%) and FD (55%) and small amounts of variation in %E from sugar and %E from fat (Supplemental Table 1). An increasing *z* score for DP1 was associated with greater %E from sugar, %E from fat, and DED and lower FD (**Figure 1**, **Supplemental Table 3**). This pattern was characterized by higher intakes of energy-dense foods, including confectionery and chocolate, cakes and biscuits, SSBs, and low-fiber bread, and lower intakes of fruit, vegetables, and high-fiber bread and cereals and was similar to the DP we identified in 2012 (**Figure 2**).

The second major DP (DP2) at 7 y of age was a new pattern not previously identified in this cohort. Response variable weights showed that DP2 was more strongly correlated with %E from sugar (0.77) but was negatively correlated with %E from fat (−0.62) and DED (−0.13) and not associated with FD (−0.01) (Supplemental Table 1). DP2 explained the majority of variation in %E from sugar (60%), modest variation in %E from fat (39%), and little to no variation in DED and FD (Supplemental Table 1). An increasing *z* score for DP2 was associated with increasing %E from sugar and decreasing %E from fat and DED (Figure 1, Supplemental Table 3). However, %E from sugar and %E from fat intakes associated with DP2 were high: in the top quintile for DP2, the mean %E from sugar was 23.7% (well above the recommended 10%) and the mean %E from fat was 31.7%. DP2 was strongly characterized by high intakes of sugary foods including SSBs, fruit juices, and ready-to-eat breakfast cereals (low-fiber breakfast cereals) and low intakes of whole milk, margarines and oils, cheese, and crisps (Figure 2). The factor loadings for DP1 and DP2 were comparable at 7, 10, and 13 y of age (available from the authors on request).

Higher *z* scores for DP1 (high in sugar, fat, and energy density and low in fiber) were longitudinally associated with a greater FMI *z* score at a later time point (**Table 1**). A 1-SD unit increase in *z* score for DP1 between follow-ups was associated with a 0.04-SD increase in FMI (95% CI: 0.01, 0.07), independent of dietary misreporting, physical activity, and maternal social class (Table 1). There was a weak interaction between DP1 and age in relation to FMI *z* score (β = −0.002, *P* = 0.08), suggesting that this association may slightly diminish with increasing age.

**TABLE 1:** Prospective associations between DP *z* scores and FMI *z* scores between 7 and 15 y of age in the ALSPAC cohort^1^

	β (95% CI)^2^	*P*^3^
Model 1^4^ (*n* = 6772)		
DP1	0.04 (0.01, 0.07)	0.017
DP2	−0.02 (−0.06, 0.01)	0.17
Model 2^5^ (*n* = 5852)		
DP1	0.04 (0.01, 0.07)	0.023
DP2	−0.01 (−0.05, 0.03)	0.54
Model 3^6^ (*n* = 4729)		
DP1	0.04 (0.01, 0.08)	0.028
DP2	−0.03 (−0.07, 0.02)	0.22

1 ALSPAC, Avon Longitudinal Study of Parents and Children; DP, dietary pattern; DP1, energy-dense, high %E from free sugars, high %E from total fat and low-fiber dietary pattern; DP2, non–energy dense, high %E from free sugars, and low %E from total fat dietary pattern; FMI, fat mass index.

2 Estimated mean change in FMI *z* score associated with a 1-SD increase in DP *z* score between dietary assessments.

3 *P* value for *z*-test: estimating the probability of rejecting the null hypothesis (β= 0) when it is true.

4 Model 1: generalized estimating equation regressing previous DP *z* score on FMI *z* score, adjusting for age, sex, and dietary misreporting.

5 Model 2: adjusted as in model 1 plus for physical activity.

6 Model 3: adjusted as in model 2 plus for maternal social class.

To assist interpretation, we estimated that an increase of a 1-SD unit in *z* score for DP1 (high in sugar, fat, and energy density and low in fiber) could be achieved by replacing a small serving of fruit equivalent to 60 g (e.g., 3 apricots, 0.5 cups grapes, or 5 medium strawberries) with an average serving of cake (90 g) while keeping all other food intakes constant. In this population of growing teenagers, a change in FMI is a function of change in FM and change in height. An increase of 0.04-SD units in FMI *z* score could be achieved by a gain in FM of 3.0 kg and a concurrent increase in height of 1.9 cm between the ages of 13 and 15 y (based on data from a girl in our data set). The mean (±SD) change in FM for all girls between 13 and 15 y of age was 2.48 ± 3.50 kg and the change in height was 2.78 ± 2.17 cm. Although in this example the change in FMI did not result in a shift from normal weight to overweight or obese status [BMI (in kg/m^2^) shifted from 21.7 at 13 y to 22.8 at 15 y], the impact of change in FM is dependent on baseline FM. Importantly, this change in FMI and FM represents the increase between 13 and 15 y of age only; small increases in adiposity tend to accumulate over time and may eventually lead to clinically significant weight gain.

**FIGURE 3:**
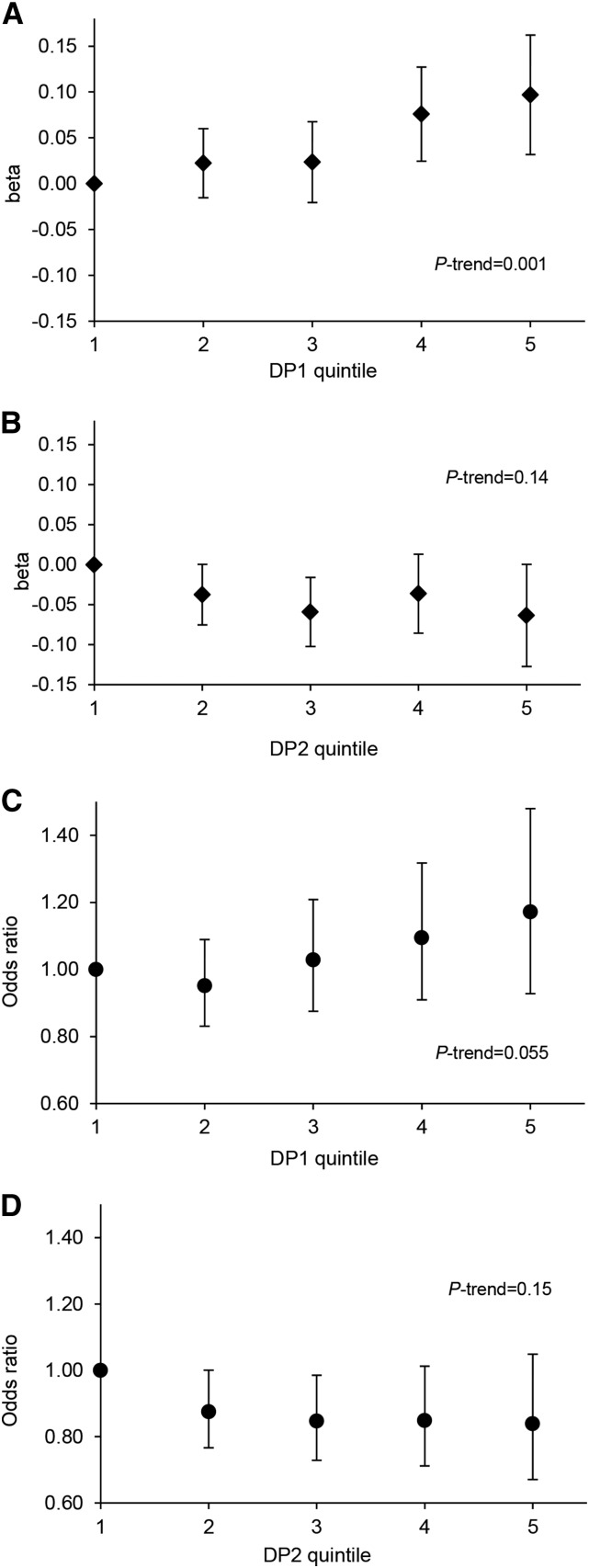
Quintiles of DP1 and DP2 *z* scores in relation to FMI *z* score (A, B) and odds of excess adiposity (C, D) between 7 and 15 y of age in the ALSPAC cohort. (A, B) Plotted values (diamonds) represent the mean increase in FMI *z* score (β-coefficients and 95% CIs) associated with a quintile increase in DP *z* score between follow-ups, relative to the lowest quintile, adjusted for age, sex, dietary misreporting, physical activity, and maternal social class. (C, D) Plotted values (circles) represent the odds of excess adiposity (ORs and 95% CIs) associated with a quintile increase in DP *z* score between follow-ups, relative to the lowest quintile, adjusted for age, sex, dietary misreporting, physical activity, and maternal social class. *P*-trend values were derived from modelling DP quintiles as continuous variables. DP1 median *z* score in each quintile: −1.37 (Q1), −0.47 (Q2), 0.06 (Q3), 0.56 (Q4), and 1.28 (Q5); DP2 median *z* scores in each quintile: −1.07 (Q1), −0.47 (Q2), −0.05 (Q3), 0.39 (Q4), and 1.14 (Q5). ALSPAC, Avon Longitudinal Study of Parents and Children; DP, dietary pattern; DP1, energy-dense, high %E from free sugars, high %E from total fat, and low-fiber dietary pattern; DP2, non–energy dense, high %E from free sugars, and low %E from total fat dietary pattern; FMI, fat mass index; Q, quintile; %E, proportion of total energy intake.

With each quintile increase in DP1 *z* score there was an increasing trend in FMI *z* score (*P* = 0.001) (**Figure 3**). Individuals in the top quintile of DP1 *z* scores had a 0.10-SD (95% CI: 0.03, 0.16) higher FMI *z* score relative to their peers in the lowest quintile (Figure 3).

The odds of excess adiposity (having an FMI in the top 20th percentile) was positively associated with DP1. A 1-SD unit increase in DP1 *z* score between follow-ups was associated with a 12% higher odds of excess adiposity (OR: 1.12; 95% CI: 1.00, 1.25) (**Table 2**). This association was stable after adjustment for dietary misreporting and physical activity (OR: 1.14; 95% CI: 1.0, 1.29) but was slightly attenuated after additional adjustment for maternal social class (OR: 1.11; 95% CI: 0.97,1.28). The odds of excess adiposity among children in the top compared with the bottom quintile of DP1 *z* score after adjustment was 1.17 (95% CI: 0.93, 1.48) (Figure 3). No interactions between DP1 *z* score and age were observed in relation to excess adiposity.

**TABLE 2:** Prospective associations between DP *z* scores and risk of excess adiposity between 7 and 15 y of age in the ALSPAC cohort^1^

	OR (95% CI)^2^	*P*^3^
Model 1^4^ (*n* = 6772)		
DP1	1.12 (1.01, 1.25)	0.038
DP2	0.95 (0.83, 1.08)	0.42
Model 2^5^ (*n* = 5852)		
DP1	1.14 (1.00, 1.29)	0.045
DP2	0.96 (0.83, 1.11)	0.55
Model 3^6^ (*n* = 4729)		
DP1	1.11 (0.97, 1.28)	0.14
DP2	0.92 (0.78, 1.09)	0.34

1 ALSPAC, Avon Longitudinal Study of Parents and Children; DP, dietary pattern; DP1, energy-dense, high %E from free sugars, high %E from total fat and low-fiber dietary pattern; DP2, non–energy dense, high %E from free sugars, and low %E from total fat dietary pattern; FMI, fat mass index.

2 Estimated odds of excess adiposity associated with a 1-SD increase in DP *z* score between dietary assessments.

3 *P* value for z-test: estimating the probability of rejecting the null hypothesis (OR = 0) when it is true.

4 Model 1: generalized estimating equation (logistic) regressing previous DP *z* score on excess adiposity (FMI *z* score >80th percentile), adjusted for age, sex, and dietary misreporting.

5 Model 2: adjusted as in model 1 plus for physical activity.

6 Model 3: adjusted as in model 2 plus for maternal social class.

DP2 (high in sugar and low in fat and energy density) was not associated with FMI *z* score (Table 1, Figure 3) or odds of excess adiposity (Table 2, Figure 3). Adjustment for pubertal development made little difference in any of the reported effect sizes but reduced the number of participants included in the models significantly and was therefore not included in the final reported models.

## Discussion

This longitudinal analysis extends our previous work in a large population-based cohort of children by identifying and contrasting 2 major DPs characterized by their free sugar, fat, energy-density, and fiber content and their relations with adiposity. Both DPs were high in free sugars, but they differed somewhat in fat intake and, to lesser degree, in energy density. Only the energy-dense DP (DP1) that was high in both sugar and fat was longitudinally associated with a greater FMI and increased risk of excess adiposity between 7 and 15 y of age. The lack of association for the DP that was high in sugar but lower in fat and energy density (DP2) suggests that a DP mostly characterized by high sugar intake is not a predictor of adiposity in this cohort, whereas the positive associations for a DP high in both fat and sugar (DP1) highlight their joint importance as a determinant of obesity in childhood.

The magnitude of associations observed between DP1 and adiposity is similar to that observed between our original energy-dense, high-fat, low-fiber DP and adiposity (0.04-SD unit increase in FMI *z* score; 95% CI: 0.01, 0.07) (9). This is likely because our original DP and DP1 from the present analysis (although derived by using different response variables) are similar in energy density, %E from fat, %E from sugar, and FD profiles. Interestingly, we did not observe a common DP in this cohort that was high in fat and low in sugar; both of the observed major DPs were high in free sugars, indicating that sugar is a prevailing component of the diets of these children.

Meeting population targets for intakes of free sugars (<10 %E) and fat (<30 %E) is challenging and it may be preferable to adopt a food-based approach to translate nutrient prescriptions into public health guidance for consumers. We examined DPs rather than individual nutrients or foods to gain insight into food-based DPs associated with obesity and to take account of several nutrients that may modulate the risk of obesity, and their potential interactions. This analysis allowed us to identify key food groups that, together, comprise a DP associated with greater adiposity. Those food groups likely to confer a greater adiposity risk (having a positive factor loading on DP1) included confectionery and chocolate, cakes and biscuits, SSBs, crisps, low-fiber breads, and low-fiber breakfast cereals. Food groups likely to confer a lower risk (negatively loaded on DP1) included fruits, vegetables, high-fiber breakfast cereals, and high-fiber breads.

Two meta-analyses of randomized controlled trials and observational studies highlighted roles for dietary fat and free sugars in weight gain (5, 6). The 2013 meta-analysis confirmed a link between SSBs and body fatness but did not find a consistent association between free sugar intake per se and adiposity in children (6). This is possibly because the limited number of randomized controlled trials in children focused on advice to reduce SSB consumption and these reported poor compliance (6). Furthermore, only 3 of the 21 cohort studies included in the meta-analysis examined sugar intake as added sugar or sucrose intake in relation to adiposity and these reported mixed findings (21–23). The inconsistencies may also result from these studies having a focus on SSBs or macronutrients only while ignoring other dietary factors and the diet as a whole. We are not aware of any other studies to date that have examined food-based empirical DPs characterized by high sugar and fat intakes with the use of the RRR method in children or adults. However, an Australian longitudinal study of 4164 children aged 4–5 y old reported that intakes of SSBs and high-fat foods were positively associated with a higher BMI *z* score 6 y later (24).

This analysis has several strengths. In using the RRR method, we applied a hypothesis-driven approach that identified patterns in food intake explaining the nutrients of most interest: free sugars, fat, fiber, and DED. The use of 3-d food diaries at each follow-up (7, 10, and 13 y) provides a high amount of detail on usual dietary intake and allowed us to examine DPs throughout childhood and adolescence rather than a single time point only. However, there is no error-free method for assessing usual dietary intake. Underreporting of EI in self-reports is common among adolescents (25) and overweight respondents and can lead to biased diet-disease observations (26). For this reason, we attempted to control for dietary misreporting in our statistical analyses. Notwithstanding concerns about individuals’ underreporting of EI, it is clear that food diaries reveal substantial intraindividual variation in the proportions of different types of foods and drinks consumed, which can be exploited in DP analyses. We were also able to adjust for objectively measured physical activity, and this had minimal effect on the associations.

This study was conducted in a geographically defined area of the United Kingdom; although the cohort was representative of the United Kingdom population at recruitment, cohort attrition led to the current “enrolled sample” having greater representation from children with a higher level of educational achievement and higher family income (11), which may limit generalizability. However, the large number of children and the number of repeated measurements included in this analysis is a major strength. Furthermore, by using longitudinal statistical models (GEEs), all of the available data points were analyzed rather than limiting and potentially biasing the analysis to only those children who completed every follow-up.

In conclusion, a DP high in both fat and sugar is longitudinally associated with greater adiposity between 7 and 15 y of age in this cohort. In contrast, a DP similarly high in %E from free sugars but lower in %E from total fat and energy density was not associated with adiposity. Although our analysis does not support the contention that sugar has a unique role in the etiology of obesity, sugar needs to be considered as part of an overall DP, and it is clear that sugar and fat are key features of DPs that are linked to excess weight gain. Public health interventions to limit the consumption of sugar, together with dietary fat, and to boost fiber are urgently needed.

## Footnotes

9 Abbreviations used: ALSPAC, Avon Longitudinal Study of Parents and Children; DED, dietary energy density; DP, dietary pattern; EER, estimated energy requirement; EI, energy intake; FD, fiber density; FM, fat mass; FMI, fat mass index; GEE, generalized estimating equation; RRR, reduced rank regression; SSB, sugar-sweetened beverage; %E, proportion of total energy intake.
